# Psychosocial interventions for premature ejaculation

**DOI:** 10.1590/S1516-31802012000200013

**Published:** 2012-04-03

**Authors:** Tamara Melnik, Stanley Althof, Álvaro Nagib Atallah, Maria Eduarda dos Santos Puga, Sidney Glina, Rachel Riera

## Abstract

**BACKGROUND:**

Premature ejaculation (PE) is a very common sexual dysfunction among patients, and with varying prevalence estimates ranging from 3% to 20%. Although psychological issues are present in most patients with premature PE, as a cause or as a consequence, research on the effects of psychological approaches for PE has in general not been controlled or randomised and is lacking in long-term follow up.

**OBJECTIVE:**

To assess the efficacy of psychosocial interventions for PE.

**CRITERIA FOR CONSIDERING STUDIES FOR THIS REVIEW:**

Trials were searched in computerized general and specialized databases, such as: MEDLINE by PubMed (1966 to 2010); PsycINFO (1974 to 2010); EMBASE (1980 to 2010); LILACS (1982 to 2010); the Cochrane Central Register of Controlled Trials (Cochrane Library, 2010); and by checking bibliographies, and contacting manufacturers and researchers.

**SELECTION CRITERIA:**

Randomised or quasi-randomised controlled trials evaluating psychosocial interventions compared with different psychosocial interventions, pharmacological interventions, waiting list, or no treatment for PE.

**DATA COLLECTION AND ANALYSIS:**

Information on patients, interventions, and outcomes was extracted by at least two independent reviewers using a standard form. The primary outcome measure for comparing the effects of psychosocial interventions to waiting list and standard medications was improvement in IELT (i.e., time from vaginal penetration to ejaculation). The secondary outcome was change in validated PE questionnaires.

**MAIN RESULTS:**

In one study behavioral therapy (BT) was significantly better than waiting list for duration of intercourse (MD (mean difference) 407.90 seconds, 95% CI 302.42 to 513.38), and couples’ sexual satisfaction (MD -26.10, CI -50.48 to -1.72). BT was also significantly better for a new functional-sexological treatment (FS) (MD 412.00 seconds, 95% CI 305.88 to 518.12), change over time in subjective perception of duration of intercourse (Women: MD 2.88, 95% CI 2.06 to 3.70; Men: MD 2.52, CI 1.65 to 3.39) and couples’ sexual satisfaction (MD -25.10, 95% CI -47.95 to -2.25), versus waiting list.

**AUTHORS’ CONCLUSIONS:**

Overall, there is weak and inconsistent evidence regarding the effectiveness of psychological interventions for the treatment of premature ejaculation. Three of the four included randomised controlled studies of psychotherapy for PE reported our primary outcome (Improvement in IELT), and the majority have a small sample size. The early success reports (97.8%) of Masters and Johnson could not be replicated. One study found a significant improvement from baseline in the duration of intercourse, sexual satisfaction and sexual function with a new functional-sexological treatment and behavior therapy compared to waiting list. One study showed that the combination of chlorpromazine and BT was superior to chlorpromazine alone. Randomised trials with larger group samples are still needed to further confirm or deny the current available evidence for psychological interventions for treating PE.

This is the abstract of a Cochrane Review published in the Cochrane Database of Systematic Reviews (CDSR) 2011, Issue 08, DOI: 10.1002/14651858.CD008195.pub8 (http://www.thecochranelibrary.com). For full citation and authors’ details, see reference 1.

For Latin America and the Caribbean, the full text is freely available from: http://onlinelibrary.wiley.com/doi/10.1002/14651858.CD008195.pub2/abstract;jsessionid=5D54BE1BCB5C03E8FF98E3B160A58088.d03t01
